# Folic Acid supplementary reduce the incidence of adenocarcinoma in a mouse model of colorectal cancer: microarray gene expression profile

**DOI:** 10.1186/1756-9966-30-116

**Published:** 2011-12-29

**Authors:** Yan-Wei Lin, Ji-Lin Wang, Hui-Min Chen, Yan-Jie Zhang, Rong-Lu, Lin-Lin Ren, Jie Hong, Jing-Yuan Fang

**Affiliations:** 1 Division of Gastroenterology and Hepatology, Shanghai Jiao-Tong University School of Medicine Renji Hospital, Shanghai Institute of Digestive Disease; Key Laboratory of Gastroenterology & Hepatology, Ministry of Health (Shanghai Jiao-Tong University). 145, Middle Shandong Rd, Shanghai, 200001, China

## Abstract

**Background:**

Whether Folic acid is a potential drug that may prevent the progression of colorectal carcinoma and when to use are important healthy issues we focus on. Our study is to examine the effect of folic acid on the development of the CRC and the optimal time folic acid should be provided in a mouse-ICR model induced by 1, 2-Dimethylhydrazine. Also, we investigated the gene expression profile of this model related to folic acid.

**Method:**

Female ICR mouse (n = 130) were divided into 7 groups either with the treatment of 1, 2-Dimethylhydrazine (20 mg/kg bodyweight) weekly or folic acid (8 mg/kg bodyweight) twice a week for 12 or 24 weeks. Using a 4 × 44 K Agilent whole genome oligo microarray assay, different gene expression among groups (NS, DMH, FA2, FA3) were identified and selected genes were validated by real-time polymerase chain reaction.

**Results:**

Animals with a supplementary of folic acid showed a significant decrease in the incidence, the maximum diameter and multiplicity of adenocarcinomas (*P *< 0.05). Furthermore, there were fewer adenomas or adenocarcinomas developed in the group of folic acid supplementation in pre-adenoma stage compared to group of post-adenoma stage. Meanwhile, about 1070 genes that were changed by 1, 2-Dimethylhydrazine can be reversed by folic acid and 172 differentially genes were identified between the groups of pre- and post- adenoma stage using microarray gene expression analysis.

**Conclusion:**

Our study demonstrated that folic acid supplementary was significantly associated with the decrease risk of CRC. And the subgroup of providing folic acid without precancerous lesions was more effective than that with precancerous lesions.

## Introduction

It is known that colorectal cancer (CRC) is one of the most common cancers especially in western countries, referred to a multiple process, multiple factors with high recurrence and high mortality [1]. Chemoprevention methods for CRC have obtained increasing attention as surgery and chemotherapy strategies perform little function once diagnosed to be tumor that invades the muscularis propria. Also, the Non-steroidal anti-inflammatory drugs (NSAIDs), such as COX-2 inhibitors, are not always successful, and may have some harmful side-effects [2]. Generally, clinical trials require at least 3-5 years follow up and a large number of patients are difficult to control their lifestyles such as smoking and wine intake which may affect the incidence of cancer [3,4]. Therefore, we choose animal model induced by chemistry drugs 1, 2-dimethylhydrazine (DMH) to simulate the formation of CRC. As azoxymethane (AOM) or 1, 2-dimethylhydrazine (DMH)-induced colon carcinogenesis in mice or rat have been identified as a useful tool [5-9]. In the previous study, we have successfully induced CRC in this model using ICR mice [9].

Folic Acid (FA) is one kind of water-solubility vitamin, which has been believed to be chemo-preventive agent that can provide methy-group to DNA thus impact DNA synthesis and DNA methylation [10]. Abbreviations in DNA synthesis often lead to DNA mutation, DNA strand break and the impairment of DNA repair, which finally result in cancer formation [11].

However, there are many conflicting data about whether FA can inhibit or promote colorectal adenoma (CRA) from clinical or preclinical studies. Epidemiologic study shows that folic acid is significant associated with lower risk and not related to the increased risk of colorectal cancer, supporting folic acid as a protective role for colon mucosa [12,13], including several large prospective studies in 99,523 participants in the American Cancer Prevention Study II (CPS-II) Nutrition Cohort [12] and in double-blind, randomized clinical trial (RCT) conducted by 9 clinical centers incorporating 1091 participants for 3 years follow up [13]. However, the Aspirin/Folate Polyp Prevention Trial demonstrated that about 67% increased risk of advanced lesions with high malignant potential, and an increased risk of having multiple adenomas among the folic acid supplementation group by providing folic acid for 6 years at 1 mg/d [14]. While other researches have reported that there is no relation or positive association between folic acid supplementation and the risk of colon adenoma [15]. Therefore, a systematic description from RCTs investigating the relation between folic acid supplementation and the risk of colorectal cancer was conducted by many groups. One recent Meta-analysis data revealed that folic acid supplementary for 3 years had no effect on the adenoma recurrence while had an increased risk of adenoma lesion for those who received folic acid over 3 years [16]. Another Meta-analysis divided the RCTs into different groups including populations with a history of adenoma and with an-average risk populations. They concluded that the evidence that folic acid was effective in the chemoprevention of colorectal cancer was not enough in both populations [17].

Further, many researchers consider that the role of folic acid might be two-sided, that is to prevent in early phage of adenoma formation and to promote in late stage depending on the time of folic acid administration. Preclinical studies have suggested that folic acid may only protect against the development of CRC in normal colon-rectum rather in mucosa with an Aberrant Crypt Foci (ACF) status [18], which is the earliest pre-neoplastic lesion that can be recognized based on the morphology and pathology features [19,20], and the results were consistent with an AOM induced rat model of CRC [21]. These experiments demonstrated that folic acid had dual effects on the development of CRC depending on the timing and dose of the intervention of folic acid [11] However, the function that folic acid may perform to the exiting adenomas in chemicals induced mouse model and the possible mechanism is still un-established now.

In this study, we use ICR mice with 1, 2-Dimethylhydrazine (DMH) interfered models to analyze the impact of folic acid on different timing courses during the processes of CRC. We have previously demonstrated that 4 weeks old ICR mice given high dosage (8 mg/ml) folic acid for 20 weeks have much more apparent effects to prevent CRC incidence than low folic acid dosage (4 mg/kg bodyweight) group using DMH-induced mice model [9]. Therefore, to investigate the role of folic acid in the process of adenoma formation, we use the dose of 8 mg/kg bodyweight. Meanwhile, we inferred that the occurrence of adenoma may take place at the course of 12 weeks based on the performance of mice in previously study, so we designed the 12^th ^week as the division of the prophase or advanced stage of CRC. The study is expected to guide the clinical application of folic acid and to identify the mechanism of folic acid in a microarray gene expression profile.

## Materials and methods

### Ethics Statement

Our study had been approved by Animal Care and Use Committee of Shanghai Jiao-Tong University School of Medicine Ren-Ji Hospital, Shanghai, China (approval ID: 2007-036. All animal procedures were performed according to guidelines developed by the China Council on Animal Care and protocol approved by Shanghai Jiao-Tong University School of Medicine Ren-Ji Hospital, Shanghai, China.

### Chemicals

1, 2-Dimethylhydrazine (DMH) and Folic acid (FA, F8758) were obtained from Sigma Chemical Co. (St. Louis, MO, USA). The PH value of DMH is adjusted with NaHCO3 to 6.5-7.0. DMH was dissolved with Normal saline and Folic Acid with drinking water.

### Experimental animals

**Figure 1:**
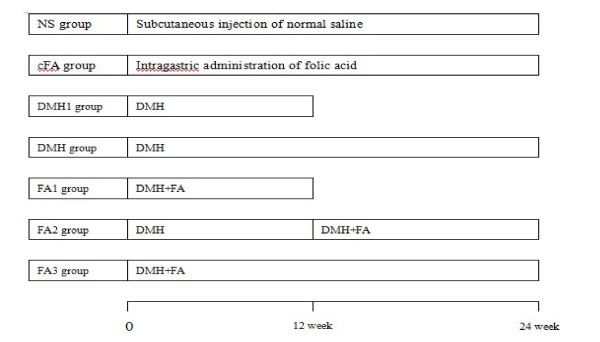
**Groups of this study**. NS group = Subcutaneous injection of physiological saline; DMH1 group = Subcutaneous injection of DMH for 12 weeks; DMH group = Subcutaneous injection of DMH for 24 weeks; cFA (control Folic Acid) = only intragastric administration of folic acid without DMH injection; FA1 = intragastric administration of folic acid with DMH injection for early 12 weeks; FA2 = intragastric administration of folic acid with DMH injection for later 12 weeks; FA3 = intragastric administration of folic acid with DMH injection for 24 weeks.

130 females, 4 weeks old ICR mice (weight, 18-20 g; grade, specific pathogen-free (SPF)) were bought from the Chinese Academy of Sciences (Shanghai, China). The mice were raised at constant temperature of 22°C with a relative humidity of 60% and 12-hour light/dark cycles; they were supplied a standard laboratory diet and drinking water. These 130 mice were randomly divided into 7 groups (Figure 1): NS group = 20 (Subcutaneous injection of physiological saline); DMH1 group = 20(Subcutaneous injection of DMH for 12 weeks); DMH group = 20 (Subcutaneous injection of DMH for 24 weeks); Cfa (control Folic Acid) = 10 (only intragastric administration of folic acid without DMH injection; FA1 = 20 (intragastric administration of folic acid with DMH injection for early 12 weeks); FA2 = 20(intragastric administration of folic acid with DMH injection for later 12 weeks); FA3 = 20 (intragastric administration of folic acid with DMH injection for 24 weeks). DMH was given subcutaneous injection once a week at the dosage of 20 mg/kg and folic acid was given by intragastric administration twice a week. All mice were weighted once a week. At the 12^th ^weeks after DMH injection, 10 of NS and groups of DMH1, FA1 were killed and the conditions of organs were recorded. The mass number and size were assessed using a micrometer. Some fresh colon and rectal tissues were maintained immediately in liquid nitrogen, and others include liver or gastric tissues were fixed in formalin solution and embedded in paraffin blocks for pathological analysis. At the end of 24^th ^weeks, all remaining mice were killed using the same methods.

### Histological Analysis

For pathology analysis, 4-μm thick sections of formalin-fixed, paraffin-embedded tissues were prepared. After hematoxylin and eosin staining, the sections of each tumor were examined under a light microscope (Olympus, Japan).

### RNA extraction and Real-time polymerase chain reaction labeling, hybridization, and analysis

Total RNAs from normal colonic mucosa of all groups were got using TRIzol (Invitrogen, USA) according to manufacturer's instruction. RNA content and purity were measured using Nanodrop ND-1000, and denaturing gel electrophoresis was performed. Next, Reverse transcription and quantification of gene expression was performed according to the manufacture's introduction (Takara). We used 18s as an internal control in Real- time PCR. Next, 3 samples of non-tumor colon of the group of NS, DMH, FA2, FA3 were amplified and labeled with the Agilent Quick Amp labeling kit and hybridized using Agilent whole genome oligo microarray (Agilent Technologies, Palo Alto, CA, USA) by using Agilent SureHyb Hybridization Chambers. Then, the processed slides were scanned with the Agilent DNA microarray scanner according to the settings provided by Agilent Technologies.

The microarray data sets were normalized by Agilent GeneSpring GX software (version 11.0) using the Agilent FE one-color scenario (mainly median normalization). Differentially expressed genes were identified via the fold-change (FC) and p values of the *t*-test. Differentially expressed genes are identified to have an FC of ≥ 1.5 and a p value of ≤ 0.05 between two groups. Functional differences of the differentially expressed genes was analyzed using the Gene Ontology (GO; http://www.geneontology.gov/).

### Statistical analysis

The results of the animal experiments and real-time PCR were analyzed using SAS 9.2 software (SAS Institute Inc. USA) with data presented in the forms of means ± SD. Student's *t*-test was used to compare values between two independent groups. Differences were considered to be significance when p < 0.05.

## Results

### Results of Animal Experiment

In the 12^th ^week, 2 of 20 mice in DMH group were discovered average 2 × 3 mm adenoma, while there is none in FA1 and NS groups. Thus, the 12^th ^week after DMH treatment might be considered to be the pre-stage that adenomas formed in DMH-induced model.

**Figure 2:**
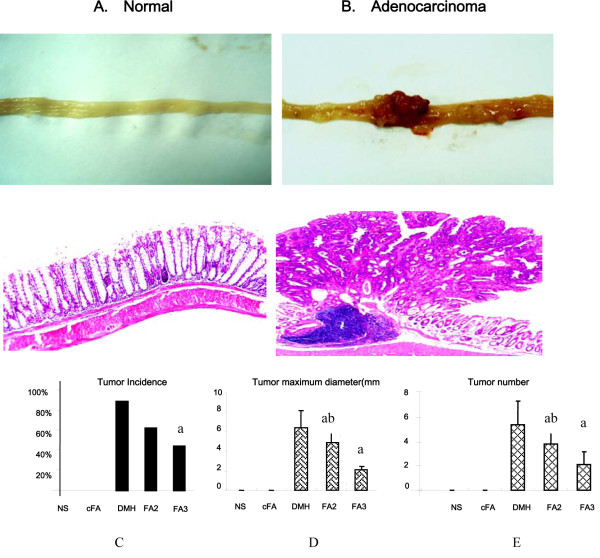
**Main results of the animal experiment after sacrificed at the 24 weeks**. A. The morphology of normal colon in macroscopic observation (Upper) and microscopy (HE stained) (Lower). Neither signs of injury nor tumor were found in NS group and cFA group. B. The morphology of colon adenocacinoma in macroscopic observation (Upper) and microscopy (HE stained) (Lower). C. The incidences of DMH-induced colorectal tumor in different groups. DMH group is 90%, which is much higher than any other groups such as FA2, FA3 which are 63%, 45% respectively. Meanwhile, there is none in NS and cFA group. D. Maximum diameter of tumor among the 5 groups (NS, cFA, DMH, FA1 and FA2). E. Tumor number in mice among the above 5 groups. (a: P < 0.05, FA3 and FA2 compared to DMH group; b: P < 0.05, FA2 compared to FA3 group)

We have successfully induced CRC in the animal model with injection DMH for 24 weeks, which were identified as adenocarcinoma by histology analysis (Figure 2A, B). Figure 1 shows mainly results of the experiment. We can see that the incidence of DMH-induced group is 90%, much higher than any other groups such as FA2, FA3, which are 63%, 45% respectively (Figure 2C). There is significant difference between groups of FA3 and DMH but not between FA2 and DMH groups. However, the multiplicity and the size of the maximum masses in FA2 and FA3 groups are much smaller compared to the DMH group (5.39 ± 1.97, 6.44 ± 1.72 mm), indicating that folic acid may prevent the growth of adenomas.

Although the incidence in FA2 is higher than FA3, no significant difference was seen between them (63% vs 45%). However, the number and the maximum diameter of the masses in FA3 group (2.11 ± 1.05, 2.11 ± 0.60 mm) showed a significant smaller than FA2 group (3.83 ± 1.11, 4.92 ± 1.24 mm), P < 0.05 (Figure 2D and Figure 2E).

There is no tumor shaped and weight loss in Folic Acid control group and the mice behavior normal, so we conclude that folic acid is safe to normal colon. Meanwhile, there was no significant difference in the growth and development of mice among DMH and FA2, FA3 groups but groups between NS and DMH. Also, a macroscopic and microscopic examination of their kidneys, stomachs, lungs, liver, and spleen showed no obvious abnormalities (data not shown).

### FA-mediated differential gene expression profile in mouse colorectal carcinogenesis model induced by DMH

With the quality control step, all twelve colonic tissues were analyzed as described in the Methods section. The microarray analysis was conducted in the NS group (3 samples), DMH group (3 samples), FA2 group (3 samples) and FA3 group (3 samples). Then we compared the gene expression levels between the samples of group NS and DMH, FA3 and DMH, FA2 and FA3.

A homogenous expression profile among the samples of each group was shown after the hierarchical clustering analysis. And when the Fold Change (FC) is set > 1.5 and the p value at ≤ 0.05, we found that the expression of 12395 genes was significantly altered in the DMH group compared to those in the NS group (see additional file 1). Together with the result of FA3 vs DMH (see additional file 2), we found that 642 genes down-regulated and 428 genes up-regulated in FA3 group compared to DMH, which may indicate that folic acid can reverse the gene expression that changed by DMH (see additional file 3). Most of these genes are metabolic-related enzymes and regulators which may perform cellular binding and enzymatic activity, involved in the biological regulation and developmental process. Other genes which are differentially expressed are closely to carcinogenesis such as cell cycle, cell invasion and apoptosis. In table 1, the most changed genes comparing FA3 group and DMH group are listed, among which are some oncogenes, for example, *Oil (*oncoprotein induced transcript 1), *Tnfrsf11b *(tumor necrosis factor receptor superfamily, member 11b), *Hmgn5 *(high-mobility group nucleosome binding domain 5) are down-regulated while tumor suppressors such as *Hnf4a *(hepatic nuclear factor 4, alpha), *Cdhr2 *(cadherin-related family member 2), *Muc2 *(mucin 2) are up-regulated. From the results of the microarray analysis, we selected 5 genes i.e., *K-ras, c-MYC, DNMT1, Tpd52, CDKN1b *for PCR confirmation because they are already considered as tumor-related genes. The primers for these genes are shown in Table 2.

**Table 1:** List of the most differentially expressed genes whose changes due to DMH treatment could be reversed by folic acid

Accession number	Gene symbol	Gene Description	Fold change	P value
**Downregulated genes**				
NM_207634	Rps24	ribosomal protein S24 (Rps24), transcript variant 2	0.002356454	2.05154E-06
NM_012052	Rps3	ribosomal protein S3 (Rps3)	0.00933479	6.38113E-06
NM_033073	Krt7	keratin 7	0.024674534	0.001286211
NM_024478	Grpel1	GrpE-like 1, mitochondrial (Grpel1)	0.029123617	3.65271E-05
NM_024243	Fuca1	fucosidase, alpha-L- 1	0.031740456	0.000162318
NM_146050	Oit1	oncoprotein induced transcript 1	0.032247549	0.001799574
NM_013614	Odc1	ornithine decarboxylase, structural 1	0.032361	4.48641E-05
NM_025431	Llph	LLP homolog, long-term synaptic facilitation (Aplysia)	0.036784284	1.18163E-06
NM_008764	Tnfrsf11b	tumor necrosis factor receptor superfamily, member 11b	0.041187965	7.03729E-05
NM_009402	Pglyrp1	peptidoglycan recognition protein 1	0.041272749	0.009299333
NM_010106	Eef1a1	eukaryotic translation elongation factor 1 alpha 1	0.041438052	7.22246E-06
NM_001008700	Il4ra	interleukin 4 receptor, alpha	0.043141894	0.000223171
NM_182930	Plekha6	pleckstrin homology domain containing, family A member 6	0.04544609	0.001545018
NM_011463	Spink4	serine peptidase inhibitor, Kazal type 4	0.045587012	0.000688366
NM_016710	Hmgn5	high-mobility group nucleosome binding domain 5	0.046928235	0.000333311
NM_016981	Slc9a1	solute carrier family 9 (sodium/hydrogen exchanger), member 1	0.052191789	5.29847E-05
NM_145533	Smox	spermine oxidase (Smox), transcript variant 2	0.053274908	6.23127E-05
NM_008305	Hspg2	perlecan (heparan sulfate proteoglycan 2)	0.056450624	0.001205571
NM_172051	Tmcc3	transmembrane and coiled coil domains 3	0.058793481	0.001122075
NM_009768	Bsg	basigin (Bsg), transcript variant 1	0.061259044	0.000407939
**Upregulted genes**				
NM_009946	Cplx2	complexin 2	1109.786672	0.000155322
NM_001039493	Plekhm3	pleckstrin homology domain containing, family M, member 3	56.2494337	0.000450001
NM_024272	Ssbp2	single-stranded DNA binding protein 2 (Ssbp2), transcript variant 2	54.215495	2.06403E-05
NM_175013	Pgm5	phosphoglucomutase 5	47.38198278	1.84156E-05
NM_008222	Hccs	holocytochrome c synthetase	39.34022581	0.000130923
NM_001033364	Cdhr2	cadherin-related family member 2	38.97741927	0.000749154
NM_023566	Muc2	mucin 2	30.63268666	0.02159023
NM_010418	Herc2	hect domain and RCC1 (CHC1)-like domain (RLD) 2	29.34751955	0.003432199
NM_008261	Hnf4a	hepatic nuclear factor 4, alpha	28.66993377	0.000234502
NM_176850	Bptf	bromodomain PHD finger transcription factor	26.66298996	0.000156324

Fold change and P values are the results comparing FA3 group and DMH group.

**Table 2:** Primer sequence for real-time pcr

Gene name	Forward sequence	Reverse sequence	Product
			length
Tpd52	tctaaagtaggaggagccaagc	gctctctgtcatctgttctgga	117
DNMT1	caagaagaaaggcaaggtcaac	cctggatgctctcaagtaggtc	212
c-Myc	atttctatcaccagcaacagcag	aacataggatggagagcagagc	137
K-RAS	tggtcctggtagggaataagtg	cccatctttgctcatcttttct	191
CDKN1b	cttgcccgagttctactacagg	agagtttgcctgagacccaat	127
Tnfrsf12a	cgaccacacagcgacttct	ccaaaaccaggaccagactaag	106
VDR	tgaaggagttcatcctcacaga	gataatgtgctgttgctcctca'	128
18S rRNA	cggacaggattgacagattgatagc	tgccagagtctcgttcgttatcg	150

However, from the analysis of microarray there are only 172 differentially genes expressed between FA2 group and FA3 group (see additional file 4). Consistent with the animal experiment that FA2 group have increase number and diameter of multiple masses, there are some tumor suppressors down-regulated in FA2 group, such as *VDR *(vitamin D receptor, FC = 0.30101), *CDX2(FC = 0.24596)*, and oncogenes up-regulated, i.e, *FN1 *(fibronectin 1, FC = 3.859909), *TNFRSF12A *(tumor necrosis factor receptor superfamily, member12a, FC = 2.515130), *NPM1*(nucleophosmin1, FC = 1.557789) that have been functional in the process of cell proliferation, cell adhesion, cell differentiation and apoptosis(see table 3). It is the first study that different genes are identified caused by the time that folic acid is provided either in the pre- or post- carcinoma stage.

**Table 3:** Partial list of the differentially expressed genes between FA2 and FA3

Accession number	Gene symbol	Gene Description	Fold change	P value
**Upregulated genes**				
NM_009758	BMPR1A	bone morphogenetic protein receptor, type 1A	2.044809816	0.015778782
NM_008722	Npm1	nucleophosmin 1	1.557789177	0.019815969
NM_022563	Ddr2	discoidin domain receptor family, member 2	3.237694059	0.036468073
NM_026653	Rpa1	replication protein A1	1.568298305	0.049492698
NM_010730	ANXA1	annexin A1	3.666236872	0.034499347
NM_009242	SPARC	secreted acidic cysteine rich glycoprotein	2.576417983	0.004456278
NM_025866	Cdca7	cell division cycle associated 7	2.483199204	0.032125313
NM_013749	TNFRSF12A	tumor necrosis factor receptor superfamily, member 12a	2.515130632	0.001750863
NM_026148	LIMS1	LIM and senescent cell antigen-like domains 1	1.897061785	0.022103283
NM_010233	Fn1	fibronectin 1	3.859908549	0.036063689
NM_133918	EMILIN1	elastin microfibril interfacer 1	2.165900048	0.018411074
NM_133721	ITGA9	integrin alpha 9	2.471522431	0.019449109
NM_022563	DDR2	discoidin domain receptor family, member 2	3.237694059	0.036468073
NM_178665	LPP	LIM domain containing preferred translocation partner in lipoma	4.202943318	0.034835063
NM_026361	PKP4	plakophilin 4	1.685566251	0.028039843
NM_010480	HSP90AA1	heat shock protein 90, alpha (cytosolic), class A member 1	1.656494408	0.029335434
NM_010135	ENAH	enabled homolog (Drosophila) (Enah), transcript variant 1	2.96541359	0.030677412
NM_013885	CLIC4	chloride intracellular channel 4	1.737725253	0.044653582
NM_010663	KRT17	keratin 17	3.435610932	0.02165621
NM_001081185	Flnc	filamin C, gamma	4.041058771	0.02814183
**Downregulated genes**				
NM_007673	Cdx2	caudal type homeobox 2	0.24596643	0.030973362
NM_145953	CTH	cystathionase	0.31273227	0.002366272
NM_008885	PMP22	peripheral myelin protein 22	0.576303226	0.031915491
NM_011146	Pparg	peroxisome proliferator activated receptor gamma	0.483425898	0.035947091
NM_138942	Dbh	dopamine beta hydroxylase	0.411709887	0.018408936
NM_020257	CLEC2I	C-type lectin domain family 2, member i	0.572216631	0.009695318
NM_010708	LGALS9	lectin, galactose binding, soluble 9	0.610346325	0.033584593
NM_011146	PPARG	peroxisome proliferator activated receptor gamma	0.483425898	0.035947091
NM_009504	VDR	vitamin D receptor	0.30101348	0.021805069
NM_015789	DKKL1	dickkopf-like 1	0.628957018	0.004386895

Fold change and P values are the results comparing FA2 group and FA3 group.

Using the GO and KEGG software, we analyzed our microarray dataset (on the basis of the results shown in additional file 3) to identify whether specific biological pathways or functional gene groups were differentially affected by the supplementary of folic acid (see additional file 5). We found that there are 63 signaling pathways including some tumor-related pathways such as Mismatch repair, focal adhesion, cell cycle and mTOR signaling pathway et al. (see additional file 6). Importantly, there are some key enzymes of metabolism pathways including fatty acid metabolism, oxidative phosphorylation decreased in FA3 group compared with DMH group, which may indicate that the decrease of the ability of the metabolism is unfavorable to tumor growth. And the most enriched pathways are shown in table 4.

**Table 4:** The most enrichment pathways related to tumorgegesis by KEGG

Pathway ID	Pathway name	Selection Count	Count	Enrichment
mmu05219	Bladder cancer - Mus musculus (mouse)	22	44	3.709033
mmu05216	Thyroid cancer - Mus musculus (mouse)	17	31	3.597993
mmu03430	Mismatch repair - Mus musculus (mouse)	13	23	3.030142
mmu05211	Renal cell carcinoma - Mus musculus (mouse)	30	77	2.524291
mmu04520	Adherens junction - Mus musculus (mouse)	29	79	2.035831
mmu04912	GnRH signaling pathway - Mus musculus (mouse)	36	104	1.939698
mmu05214	Glioma - Mus musculus (mouse)	27	74	1.892937
mmu04110	Cell cycle - Mus musculus (mouse)	46	140	1.872654
mmu05215	Prostate cancer - Mus musculus (mouse)	31	94	1.446692
mmu04150	mTOR signaling pathway - Mus musculus (mouse)	20	56	1.429803
mmu05200	Pathways in cancer - Mus musculus (mouse)	98	345	1.369825
mmu05221	Acute myeloid leukemia - Mus musculus (mouse)	21	61	1.309804

"SelectionCounts" stands for the Count of the DE genes' entities directly associated with the listed PathwayID;

"Count" stands for the count of the chosen background population genes' entities associated with the listed PathwayID;

## Discussion

In this analysis with a DMH-induced CRC model, we concluded that the supplementation of folic acid can decrease the risk of CRC and the subgroup of providing folic acid without precancerous lesions was more effective than that with precancerous lesions. Significantly, there was a reduction in the tumor mass diameter and multiplicity in folate supplementation group. Moreover, the study is consistent with many other studies either in rodent models or clinical medical researches. Recently, a study that investigated 2299 incidents and 5655 CRA in Nurses' Health Study and Health Professionals Follow-Up Study showed that folic acid intake 12-16 y before diagnosis was inversely associated with CRC and identified the latency that folic acid should be provided. However, the study didn't analyze the results that folic acid was provided after diagnosis [22]. With the same kind of chemical in a rat model of CRC, folate deficiency was found to enhance the development of neoplasia compared to the diets containing 8 mg/kg folic acid [21] but the study had no related mechanisms. However, some studies observed the opposite results. Le Leu [23] believed that folate deficiency can decrease the development of the intestinal tumors in AOM-induced SD-rat model. To this point, we think that the animal strain, experimental condition, experiment skills, folic acid manufactories, folic acid intervention time et al may contribute to these differences in varies studies. Also, there is a possibility that excessive intake of folic acid could have promoted the growth of pre-neoplastic lesions so that our study support that enteroscope should be conducted for the cases in clinical studies before incorporated.

On the other hand, there are still no significant differences in the incidence of cancers between group FA2 and FA3 even though the maximum diameter and the number of the tumor mass are significantly decreased in FA3 group. It may be due to too small number of mice or too much difference among individuals. In another respect, not all the mice had adenomas in the 12^th ^week as the incidence was only 10% among DMH1 group. So, further study should extend the number of samples to get more objective results.

Next, we use microarray gene expression profile analysis to study the mechanism of folic acid-mediated prevention of colon tumors and the difference in folic acid intervention time. To our knowledge, this is the first investigation to use microarray technology to study the role of folic acid in the prevention of CRC and the difference of folic acid intervention times.

**Figure 3:**
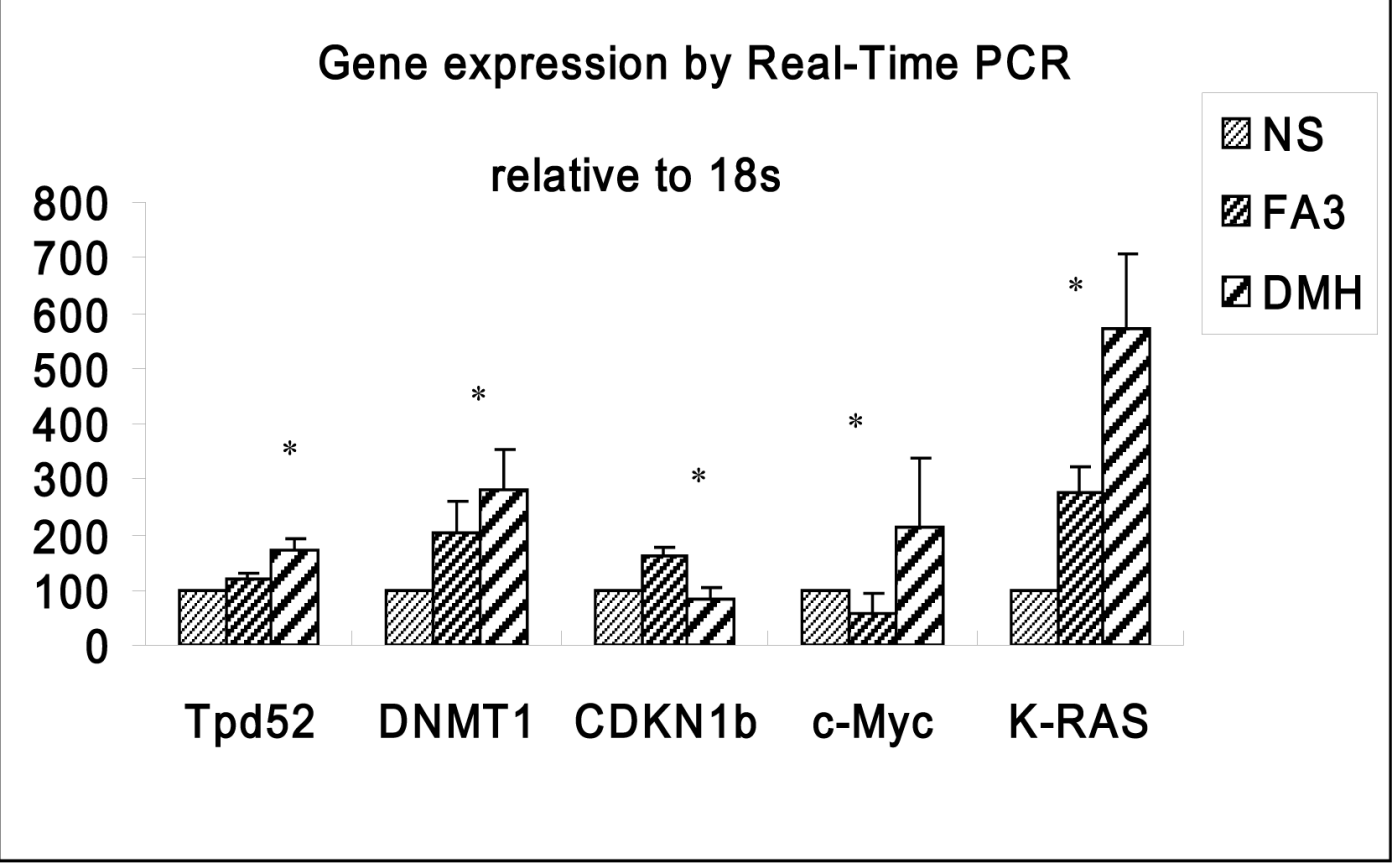
**Differentially expressed genes validated by real-time polymerase chain reaction (q-PCR)**. We used 18s rRNA as an internal control. Relative mRNA expression was calculated according to the 2^-ΔΔT ^method. Data are expressed as the mean ± SD of 10 samples. The significance of the varieties between the average values of groups DMH and FA3 was analyzed through student's t-t test. (*: P < 0.05, between FA3 and DMH group)

Firstly, when the FC was set to ≥ 1.5, 642 genes that changed with the treatment of DMH could be reversed with folic acid supplementary. We selected 5 known tumor-related genes i.e., *K-ras, c-MYC, DNMT1, Tpd52, CDKN1b *for PCR confirmation [Figure 3]. It is known that genetic alterations may contribute substantially to the pathogenesis of colon cancer. Point mutation of *K-ras *(occurring in 40% of sporadic CRCs) is an established predictor of absence of response to epidermal growth factor receptor (EGFR) -targeted agents [24,25]. Hutchins [26] reported that KRAS mutant tumors were more evenly distributed: 40% right colon, 28% left colon, and 36% rectal tumors compared to BRAF mutant tumors. Meanwhile, the relationship between Folic acid and KRAS has been studied. Some suggested that the effect of folate on rectal cancer risk is different to men and women which may depend on the status of K-ras mutation of tumors. They believed that folate intake was related to a decreased risk of G > A transitions (RR-0.08, 95% CI = 0.01-0.53) while an inversely risk of G > T and G > C transversions in tumors (RR = 2.69, 95% CI = 1.43-5.09)[27].

*CDKN1b *(cyclin-dependent kinase inhibitor 1B, FC = 7.992979) which is also known as p27 encodes a protein which belongs to the *Cip/Kip *family of cyclin dependent kinase (Cdk) inhibitor proteins [28] It is often considered as a cell cycle inhibitor protein because its major function is to control the cell cycle progression at G1 phase so that can prevent the development of cancer. Reduced p27 levels were found in different cancerous stages in hepatocelluar carcinomas [29]. Some studies demonstrated that loss of p27 expression is associated with a higher response rate to CRC chemo-therapy [30]. The p27KIP1 null (-/-) mouse shows a significant increase in cell proliferation, resulting in approximately 30% increase in mass size, multiple organ hyperplasia [31]. Together, these researches supported p27 as an important tumor suppressor and suggest that events leading to p27 upregulation may inhibit the tumor progression.

The methylation of genomic DNA in malignant cells is catalyzed by DNA methytransferases(DNMT)which include maintenance DNA methyltransferase (Dnmt1), DNMT1, de novo DNA methyltransferases (Dnmt3a and 3b), 3a/3b. DNA methylation is an important form of epigenetic that can regulate some gene expression such as *c-Myc, CDKN2a, CDH1 *and *VDR *et al [32-34]. We have seen that the expression of DNMT1 was increased in FA3 compared to DMH, which is consistent with the research that the folate - and methyl-deficient diet alters components of the DNA methylation via both transcriptional and posttranscriptional mechanisms in in livers of F344 rats [35]. Meanwhile, some methylation-related genes that are functional in carcinogenesis can also be regulated by folic acid in terms of DNA methylation [36].

Tumor necrosis factor receptor superfamily, member 12a (*Tnfrsf12a*), also known as fn14 or TWEAK-R have been implicated in a variety of pathological processes including chronic inflammation and cancers [37]. And fn14 expression is at a relative lower level in normal tissues while much higher in cancer cells or tissues [38]. Kawashima [39] reported that IL-13 may damage the mucosa of colon via the function of TWEAK and Fn14 pathway and Fn14 could aggravate intestinal inflammation in patients with UC. So the relation between fn14 and diseases might suggest fn14 and TWEAK are targets for cancer therapy [37]. In our study, *Tnfrsf12a*'s expression is 2.5 fold changes higher in FA2 group than FA3, which may be explained that the degree of colon mucosal damage in FA2 was much worse and was prone to develop to cancers compared to FA3. In this aspect, the high expression of fn14 may contribute to the growth of masses in FA2 group.

*Vitamin D Receptor *gene *(VDR) *is involved in the progress of cancers or chronic diseases [40]. Some argued that the polymorphism of *VDR *and *CDX2 *was not associated with increased risk of CRCs [41]. While others suggested that significant associations with *VDR *polymorphisms was found not in colorectal cancers but much stronger in cancers of breast, prostate and renal cell carcinomas [42]. And the association between *VDR *polymorphisms and folic acid has not been reported yet. In another respect, *VDR *is considered to be an epithelial marker in the process of Epithelial to mesenchymal transition (EMT) and thus might have a suppressive function of invasion [43]. Therefore, the expression of many tumor suppressors such as *VDR *was much lower (FC = 0.3010) compared with group FA2 and FA3, which was opposite to oncogenes.

However, there are some limitations of our study should be mentioned. First, we ignored the usage of the B Vitamins in the animal experiment, which is important in the process of Folic acid' transport and storage in liver. Therefore, Folic acid supplements may sometimes include vitamin B12 supplements with simultaneous administration of vitamin B12 [22]. However, some studies do not think there are any influences exiting with or without vitamin B12 [44]. Others even found that treatment with folic acid plus vitamin B(12) was associated with increased cancer outcomes [45]. Thus, consideration should be given to the potential value of providing with or without vitamin B12 in addition to the current mandatory folic acid supplementation.

Second, since folic acid is important in many processes of metabolism and might help to protect against the cardiovascular, mental diseases, cancer and birth defects [46]. However, we have no indicators to find other adverse effects but to observe the injury of organs in this study. Even though there are no abnormalities discovered in other organs except colon and rectum, the function of folic acid is needed to be further studied in terms of being effective to therapy.

Finally, although some similarities do exist between chemical rodent models of colon cancer and human natural CRCs, several respects of differs may also exist indeed. For example, the dose and duration of folic acid supplementation used in our study may be different from human studies. So, considering the safety of chemoprevention in clinical application, the optimal researches should be established in humans based on these findings with an initial colonoscopy before incorporated.

In summary, for the first time, our data suggest that folic acid supplementary in pre-cancerous era is much more protective than that in post-cancerous stage in a DMH induced mouse model and identify differential genes that folic acid can reversed and that between groups of pre or post-adenoma induced by folic acid using microarray gene expression profile. Not only to the reason that floate supplementation facilitates the progression of (pre)neoplastic lesions though providing nucleotide precursors to the rapidly replicating transformed cells, thus accelerating proliferation [11]. We also clarified that in gene expression profile, certain oncogenes that promote tumor growth, cell cycle, cell invasion such as *TNFRSF12A, fibronectin 1, Cdca7 *are high expressed in FA2 group compared to FA3 group while tumor suppressors are down-regulated such as *VDR, CDX2*, which may partly explain the result. However, the mechanism why folic acid provided in different phages can change these genes' expression remains to be studied.

## Competing interests

The authors declare that they have no competing interests.

## Authors' contributions

YL, JW, LR, JH carried out the molecular genetic studies, participated in the sequence alignment. YL, JW, HC, YZ participated in animal experiment. YL, JW, RL, JH, JF conceived of the study and participated in its design and coordination. YL, JW performed in the statistical analysis and drafted the manuscript. All authors read and approved the final manuscript.

## Supplementary Material

Additional file 1 **Table S1. Complete list of differentially expressed genes in the DMH group compared with the Control group**. the file contains all different genes identified by micro-array between DMH group and Control group. Click here for file

Additional file 2 **Table S2. Complete list of differentially expressed genes in the FA3 group compared with the DMH group**. the file contains all different genes identified by micro-array between FA3 group and DMH group. Click here for file

Additional file 3 **Table S3. Complete list of genes whose changes due to DMH treatment could be reversed by folic acid**. the file contains all genes that could be reserved by folic acid when treated with DMH Click here for file

Additional file 4 **Table S4. Complete list of differentially expressed genes in FA2 group and FA3 group**. the file contains complete differential genes between FA3 group and FA2 group by the micro-array Click here for file

Additional file 5 **Table S5. Complete list of the GO terms based on the genes whose changes due to DMH treatment could be reversed by folic acid**. the file contains GO terms based on the differential genes between FA3 group and DMH group by the micro-array Click here for file

Additional file 6 **Table S6. Complete list of pathways based on the genes whose changes due to DMH treatment could be reversed by folic acid**. the file contains complete pathways that could be affected by folic acid when treated with DMH Click here for file
